# Targeting glutamine metabolism as a therapeutic strategy for cancer

**DOI:** 10.1038/s12276-023-00971-9

**Published:** 2023-04-03

**Authors:** Jonghwa Jin, Jun-Kyu Byun, Yeon-Kyung Choi, Keun-Gyu Park

**Affiliations:** 1 Department of Internal Medicine, School of Medicine, Kyungpook National University, Kyungpook National University Hospital, Daegu, 41944 South Korea; 2 BK21 FOUR Community-based Intelligent Novel Drug Discovery Education Unit, Research Institute of Pharmaceutical Sciences, College of Pharmacy, Kyungpook National University, Daegu, 41566 Korea; 3 Department of Internal Medicine, School of Medicine, Kyungpook National University, Kyungpook National University Chilgok Hospital, Daegu, 41404 Korea

**Keywords:** Cancer metabolism, Cancer metabolism

## Abstract

Proliferating cancer cells rely largely on glutamine for survival and proliferation. Glutamine serves as a carbon source for the synthesis of lipids and metabolites via the TCA cycle, as well as a source of nitrogen for amino acid and nucleotide synthesis. To date, many studies have explored the role of glutamine metabolism in cancer, thereby providing a scientific rationale for targeting glutamine metabolism for cancer treatment. In this review, we summarize the mechanism(s) involved at each step of glutamine metabolism, from glutamine transporters to redox homeostasis, and highlight areas that can be exploited for clinical cancer treatment. Furthermore, we discuss the mechanisms underlying cancer cell resistance to agents that target glutamine metabolism, as well as strategies for overcoming these mechanisms. Finally, we discuss the effects of glutamine blockade on the tumor microenvironment and explore strategies to maximize the utility of glutamine blockers as a cancer treatment.

## Cancer: Targeting glutamine metabolism as potential treatment

Further insights into metabolic reprogramming by cancer cells are needed to determine if targeting glutamine metabolism could be a useful therapeutic approach. Cancer cells rely on the amino acid glutamine for growth and proliferation. Glutamine is a key nitrogen source and enables the cells to synthesize critical molecules including lipids and metabolites. Blocking glutamine metabolism may be useful in treating cancers, but care must be taken due to glutamine’s multiple roles. Keun-Gyu Park and Yeon-Kyung Choi at Kyungpook National University in Daegu, South Korea, and co-workers reviewed current knowledge of the steps involved in glutamine metabolism to identify opportunities for intervention. Cancers can evade glutamine metabolism inhibition by manipulating the tumor microenvironment and finding alternative ways to source nutrients. Drug trials have targeted aspects of glutamine metabolism with mixed success, inhibition of glutamine transporters showing promise.

## Introduction

Metabolic reprogramming, a hallmark of cancer cells, is a process by which cancer cells ensure a sufficient supply of proteins, nucleotides, and lipids to support rapid growth and proliferation^1^. The importance of cancer cell metabolism and the limitations of conventional cancer therapies (e.g., resistance to chemotherapy or radiotherapy) have prompted the development of strategies aimed at targeting this biological process^2^. Several drugs that do just that have been introduced and have shown promising results in animal studies; a few have entered clinical trials^2^. In particular, glutamine metabolism has attracted much attention as a therapeutic target because cancer cells are heavily reliant on this amino acid for growth and proliferation^3^.

Glutamine is a nonessential/conditionally essential amino acid that plays a pivotal role in clinical illness and stress conditions^4^. Glutamine in cancer cells plays critical and diverse roles by providing not only a source of nitrogen for amino acid and nucleotide biosynthesis but also a source of carbon to replenish the tricarboxylic acid (TCA) cycle and lipid biosynthesis pathways; thus, cancer cells are “addicted” to glutamine^5^. Glutamine metabolism and closely linked metabolic networks involving glutamine transporters, glutaminase, aminotransferase, and redox homeostasis are essential for cancer cell survival^6^. Targeting each step of glutamine metabolism has shown promising results in cancer treatment, prompting the discovery of druggable targets and the development of anticancer drug candidates^3^. In addition, given that immune checkpoint inhibitors are now widely used to treat cancer, the role of glutamine blockade within the tumor microenvironment (TME) has gained much attention^7^.

This review summarizes each step of glutamine metabolism in cancer cells and highlights opportunities for clinical intervention. Furthermore, we discuss resistance mechanisms and the role of glutamine blockade in the TME.

## The role of glutamine in cancer cell growth

Rapidly proliferating cancer cells take up glutamine from plasma via various amino acid transporters, and then it is converted to glutamate in the mitochondria by the two forms of glutaminase: kidney-type glutaminase (GLS)1 and liver-type GLS2^8^. Notably, GLS-mediated deamination of glutamine to glutamate is the first and rate-limiting step of glutaminolysis, making it an attractive druggable target^9^. GLS1 is overexpressed in various cancer cells, and this phenotype is associated with a higher disease stage and a poor prognosis^10^. Mechanistically, the expression of GLS1 is regulated indirectly by Myc (via repression of miR-23a and miR-23b) and mTORC1^11,12^. Unlike GLS1, GLS2 suppresses the proliferation and migration of cancer cells^13^. In hepatocellular carcinoma (HCC), GLS2 inhibited proliferation in vitro and lung metastasis in a xenograft mouse model by inhibiting the small GTPase Rac1^13^. However, several studies have shown that GLS2 is highly expressed in triple-negative basal-like breast cancer (TNBC) and metastatic lung cancer and that it confers radioresistance in advanced human cervical cancer cells, suggesting that GLS2 may reduce reactive oxygen species (ROS) levels by increasing the level of cellular reduced glutathione (GSH), NADH, or NADPH^14–16^. The confounding results regarding the function of GLS2 in cancer metabolism suggest that it may act in a context-specific manner^17^.

The TCA cycle is an essential hub for several metabolic pathways and for the interconversion of metabolites, which are renewed constantly in rapidly proliferating cancer cells^18^. Thus, replenishment of metabolic intermediates via the TCA cycle is vital to cancer cells, making them reliant on glutamine, a phenomenon called anaplerosis^8^. During anaplerosis, mitochondrial glutamate dehydrogenase 1 (GLUD1) plays a key role by catalyzing the conversion of glutamate to alpha-ketoglutarate (α-KG) and releasing ammonia, which regulates autophagy and neutralizes the intracellular pH in cancer cells^19,20^. α-KG is generated for the TCA cycle and is used for oxidative phosphorylation (OXPHOS)^21^. In addition, glutamine-derived α-KG is oxidized to succinate and fumarate, which maintain the TCA cycle in cancer cells by providing ATP, NADH, and FADH_2_ and by acting as oncometabolites^22^. Indeed, GLUD1 is overexpressed in various cancer cells, promoting epithelial-mesenchymal transition and drug resistance^23^. Mechanistically, Myc drives glutaminolysis by upregulating GLUD1 and induces a concurrent increase in the expression of GLS and SLC1A5^24^.

**Fig. 1:**
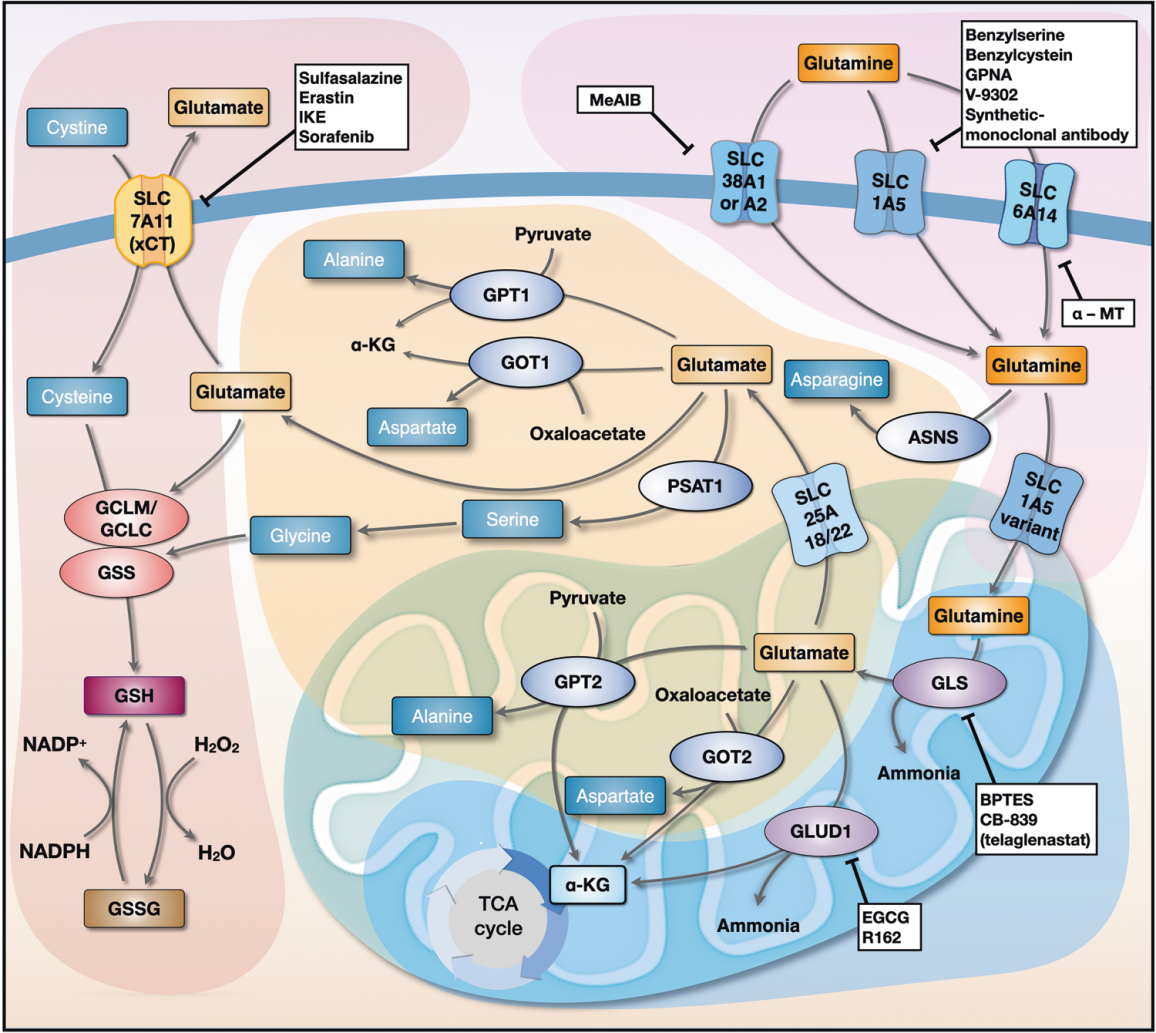
Interlinked networks involved in glutamine metabolism. Glutamine transporters (SLC1A5, SLC38A1/SLC38A2, and SLC6A14) expressed on the cell membrane transport glutamine into the cytosol. Next, the SLC1A5 variant transports glutamine to the mitochondrial matrix, where it is converted to glutamate by GLS; this is the rate-limiting step of glutaminolysis. Glutamine-derived glutamate is catalyzed into α-KG by GLUD1, GOT2, and GPT2 to release ammonia, aspartate, and alanine, respectively. Glutamine-derived glutamate in the mitochondria is also transported to the cytosol by SLC25A18/SLC25A22. GOT1, which is part of the malate-aspartate shuttle, contributes to the maintenance of redox homeostasis by converting OAA to aspartate, and GPT1 converts pyruvate to alanine. SLC7A11 transports cysteine to the cytosol in exchange for glutamate. Glutamine-derived glutamate and cysteine are ligated by GCLM/GCLC, which is in turn utilized by GSS to form GSH, which scavenges cellular ROS. Inhibitors of each step of glutamine metabolism are shown in white boxes. GLS, glutaminase; α-KG, α-ketoglutarate; GLUD1, glutamate dehydrogenase 1; GOT, glutamate oxaloacetate transaminase; GPT, glutamate pyruvate transaminase; GCLM, glutamate-cysteine ligase modifier subunit; GCLC, glutamate-cysteine ligase catalytic subunit; GSS, glutathione synthetase; GSH, reduced glutathione; ROS, reactive oxygen species; ASNS, asparagine synthetase; PSAT1, phosphoserine aminotransferase 1.

Amino acids are required by cancer cells for proliferation under genotoxic, oxidative, and nutritional stress conditions; these amino acids serve as building blocks for protein synthesis and act as substrates for glucose, lipid, and nucleic acid synthesis^25,26^. In particular, glutamine plays a vital role in this process not only by providing a carbon source to the TCA cycle but also by acting as a nitrogen source for the biosynthesis of alanine, aspartate, and serine (Fig. 1)^8^. Therefore, the role of aminotransferases such as glutamate pyruvate transaminase (GPT) and glutamate oxaloacetate transaminase (GOT) in glutamine metabolism in cancer cells has been studied extensively^8^. Regarding GPTs, cytosolic GPT1 and mitochondrial GPT2 play major roles in energy metabolism in cancer cells by providing alanine for protein synthesis and by replenishing TCA cycle intermediates^27^. Indeed, GPT2 is a significant contributor to tumorigenesis in breast cancer, glioblastoma, and KRAS-driven colorectal cancer (CRC) cells^28–31^. Because cancer cells do not take up aspartate very well, GOT fuels tumorigenesis by providing cytosolic aspartate, which is used as a precursor for protein and nucleotide synthesis and for redox homeostasis^32^. Cytosolic GOT1 and mitochondrial GOT2, which together comprise the malate-aspartate shuttle, interconvert oxaloacetate and aspartate using glutamate or α-KG as substrates^33^. Indeed, both GOT1 and GOT2 are overexpressed in KRAS-driven pancreatic ductal adenocarcinoma (PDAC) cells^34,35^. Glutamine is also required for *de novo* synthesis of asparagine via asparagine synthetase (ASNS), which is induced by either the amino acid response or the unfolded protein response pathways^36,37^. Asparagine activates mTORC1 and contributes to the biosynthesis of purines and pyrimidines, as well as to the exchange of extracellular amino acids such as histidine, aspartate, and serine^37^. Indeed, the role of ASNS in tumorigenesis and metastasis has been reported, and it is associated with poor survival in various types of breast cancer, non-small cell lung cancer (NSCLC), and sarcoma^38–40^.

## Targeting glutaminase and transaminase as a treatment for cancer

GLS, which is highly expressed in cancer cells and plays a role in cancer progression, has been investigated extensively as a druggable target^41^. Bis-2-(5-phenylacetamido-1,2,4-thiadiazol-2-yl)ethyl (BPTES), a potent orally available GLS1 inhibitor that spares GLS2, shows promising antitumor effects against human lymphoma B cells in vitro and in a xenograft mouse model^42^; it also suppresses the growth of platinum-resistant CRC and ovarian cancer cells, suggesting that combined treatments based on conventional drugs and glutamine-modulating compounds will yield clinically relevant results^43,44^. Recently, another selective inhibitor of GLS1, CB-839 (telaglenastat), showed no significant side effects in preclinical trials and is currently undergoing full clinical trials^45^. Previous studies showed that it did not significantly suppress the growth of KRAS-derived PDAC cells in vitro or in vivo because these cells mounted an adaptive metabolomic response, suggesting the importance of combined therapy for avoiding metabolic adaptation in response to GLS inhibition^45^. Thus, clinical trials are currently testing the following drugs in combination with CB-839: nivolumab as a treatment for melanoma, renal cell carcinoma (RCC), and NSCLC (clinicaltrials.gov ID: NCT02771626); everolimus for RCC (clinicaltrials.gov ID: NCT03163667); palbociclib for KRAS-derived PDAC, NSCLC and CRC (clinicaltrials.gov ID: NCT03965845); and cabozantinib for advanced RCC (clinicaltrials.gov ID: NCT03428217)^46^. Furthermore, CB-839 increased the radiosensitivity of head and neck squamous carcinoma (HNSCC) and NSCLC cells both in vitro and in a xenograft mouse model by abolishing GSH synthesis^47,48^, making it useful for concurrent chemotherapy and radiotherapy in a clinical setting.

Previous studies have shown that targeting GLUD1 inhibits the proliferation and migration of cancer cells, suggesting that GLUD1 is a druggable target for cancer therapy^23^. Epigallocatechin gallate (EGCG), an inhibitor of GLUD1 and 2, suppresses the proliferation of neuroblastoma, glioma, and CRC cells^49^. Recently, the purpurin analog R162 (an inhibitor of GLUD1) also showed promising results with respect to attenuating the proliferation of breast, NSCLC, and glioma cells in vitro and in patient-derived xenograft mouse models^23^. In addition, cotreatment of docetaxel-resistant NSCLC with docetaxel plus R162 inhibited cancer cell growth and metastasis both in vitro and in xenograft mouse models, again suggesting that combination therapy with anticancer drugs plus a GLUD1 inhibitor is an effective cancer treatment^23^.

## Role of glutamine metabolism in redox homeostasis

ROS levels are elevated persistently in proliferating cancer cells, and ROS damage DNA and cellular components; therefore, redox homeostasis plays a pivotal role in protecting cancer cells against them. Notably, GSH acts as a critical antioxidant that protects cancer cells from any form of programmed cell death (i.e., autophagy, apoptosis, necroptosis, and ferroptosis)^50–52^. Given that glutathione is a tripeptide composed of glutamate, glycine, and cysteine, glutamine-derived glutamate and cysteine need to be ligated by glutamate-cysteine ligase (GCL), which itself comprises two separately encoded proteins: a catalytic subunit (GCLC) and a modifier subunit (GCLM)^53,54^. Next, glutathione synthetase (GSS) adds glycine to the ligated glutamate-cysteine (Fig. 1)^53,54^. While glutamate and glycine are abundant in cells, cysteine is the least abundant amino acid; therefore, it must be transported into the cells by SLC7A11 (xCT) in exchange for glutamate, which implies that SLC7A11-mediated GSH biosynthesis largely relies on glutamine metabolism^55^. Intriguingly, GOT1 and malic enzyme 1 (ME1) are also associated with redox homeostasis; in PDAC, GOT1 and ME1 maintain the NADPH/NADP^+^ ratio via the malate-aspartate shuttle by maintaining GSH levels^56^. In addition, we previously showed that upon inhibition of glutamine, cancer cells reduce the amount of GSH by exporting oxidized glutathione (GSSG) out of the cell via GSSG transporters and multiple-drug resistance-associated proteins^57^ and by extracellular degradation of GSSG^58^. Given that glutamine metabolism increases the amount of GSH in cells by maintaining the NADPH/NADP^+^ ratio and by preventing export and extracellular degradation of GSSG, glutamine is the primary amino acid that controls cellular GSH homeostasis.

## Targeting redox homeostasis for cancer treatment

Approximately one-third of glutamine taken up by human fibroblast cells is exchanged for cysteine by SLC7A11^59,60^. This suggests that SLC7A11 not only plays a critical role in protein and GSH synthesis through cysteine uptake but also dictates glutamine dependence^61^. Therefore, targeting SLC7A11 is a promising therapeutic option, and its efficacy can be increased by combining it with drugs that target glutaminolysis^60^. Indeed, sulfasalazine (which inhibits SLC7A11) effectively suppresses the proliferation of glutamine-depleted TNBC in vitro and in vivo^60^. Moreover, glutamine-dependent PDAC is sensitive to the SLC7A11 inhibitor erastin, which induces ferroptosis^62,63^. However, although erastin shows antitumor effects, it has not entered clinical trials because it is poorly soluble in water, and its metabolism in vivo is unpredictable; therefore, imidazole ketone erastin (IKE) and piperazine erastin were developed (which are more soluble in water), and both show strong antitumor effects against diffuse large B-cell lymphoma (DLBCL) and fibrosarcoma^64,65^. Intriguingly, and as mentioned above, PDAC cells are dependent on GOT1 and the malate-aspartate shuttle; GOT1 knockout combined with cysteine depletion by erastin or IKE showed potent antitumor effects against these cells by reducing GSH and increasing ferroptosis^66^. In addition, sorafenib, a kinase inhibitor approved for the treatment of RCC and HCC, inhibits SLC7A11 to suppress the growth of these tumors via the induction of ferroptosis^67,68^.

## Targeting glutamine transporters as a treatment for cancer

Cancer cells require an abundant supply of glutamine from the extracellular milieu; therefore, upregulation of glutamine transporters SLC1A5, SLC38A1, SLC38A2, and SLC6A14 at the cell membrane is required (Fig. 1)^69^. Indeed, high expression of these transporters contributes to cancer cell growth and is a marker of clinically poor outcomes for patients with NSCLC, prostate cancer, breast cancer, and acute myeloid leukemia^70–73^. Thus, cancer treatment strategies have focused on pharmacological inhibition of these transporters.

### SLC1A5

SLC1A5 (ASCT2) is an obligatory sodium-dependent transporter of neutral amino acids, which are exchanged for asparagine, threonine, or serine^74^. SLC1A5 has high affinity for glutamine, particularly in an acidic environment^75^, and is thus more effective at transporting glutamine into cancer cells that thrive in acidic environments^76^. Indeed, SLC1A5 is highly expressed in various solid cancers^73,76,77^^,^ such as squamous lung cancer, in which it is responsible for approximately 50% of glutamine uptake^73^. The expression of SLC1A5 is regulated by various transcriptional regulators, including ATF4 and Myc^78,79^. In TNBC, high expression of ATF4 and Myc is associated with overexpression of SLC1A5 and indicates poor survival outcomes^77^. In addition, Myc-dependent expression of ATF4 in DLBCL cells, human colon adenocarcinoma cells, and mouse embryonic fibroblasts drives the expression of SLC1A5 during metabolic adaptation to stress conditions^73,80,81^. A recent study showed that HIF-2α-mediated overexpression of SLC1A5 variants in mitochondria plays an essential role in glutamine metabolism in pancreatic cancer cells by inducing chemotherapy resistance^82^.

Therefore, SLC1A5 is a promising druggable target^83^. Benzylserine and benzylcysteine were the first molecules found to inhibit SLC1A5 in breast and gastric cancer cells, but they are nonspecific^84,85^. L-γ-glutamyl-p-nitroanilide (GPNA) suppresses the growth of TNBC, different types of lung cancer, and neuroblastoma cells^73,77,81^. In addition, combined treatment with GPNA and a monoclonal antibody (cetuximab) targeting EGFR effectively suppressed the growth of gastric cancer cells in vitro and in vivo^86^. However, amino acid analogs are unsuitable for clinical use due to their low affinity, lack of specificity, and toxicity^87^. V-9302 (2-amino-4-bis (aryloxy benzyl) aminobutanoic acid) was originally discovered as an SLC1A5 inhibitor; it showed a 100-fold increase in potency over GPNA and attenuated the growth of cancer cells, including HCC, CRC, lung cancer, and breast cancer cells^83^. Recent studies have shown that synthetic monoclonal antibodies specific for SLC1A5 (i.e., KM4008, KM4012, and KM4018) are an effective therapeutic option for suppressing glutamine-dependent growth of CRC cells, but their efficacy in other cancer cells remains unclear^88^. Despite the significance of SLC1A5 in some cancer cells, there are few specific and effective SLC1A5-inhibiting drugs^89^.

### SLC38A1 and SLC38A2

SLC38A1 (SNAT1) and SLC38A2 (SNAT2) are sodium-dependent neutral amino acid transporters that drive glutamine influx into cells^90^. SLC38A1 is overexpressed in melanoma, breast, gastric, osteosarcoma, and endometrial cancer cells, showing a close association with proliferation and migration^91–93^. SLC38A2 is highly expressed in prostate cancer, HCC, and TNBC cells, thereby contributing to tumorigenesis^94,95^. Interestingly, silencing of SLC1A5 does not suppress the proliferation of epithelial cervical cancer and osteosarcoma cells; rather, it induces an amino acid starvation response by upregulating the expression of SLC38A1, suggesting that SLC38A1 is a major importer of glutamine into these cells^90^. In addition, amino acid starvation upregulates SLC38A2 via activation of GCN2 and ATF4, which help to maintain the intracellular glutamine pool^36,90^. Therefore, strategies designed to target glutamine metabolism should consider the combined blockade of these transporters.

N-methyl-aminoisobutyric acid (MeAIB) has been investigated in studies attempting to elucidate the function of SLC38A1 and/or SLC38A2 in various cells; indeed, MeAIB exerts an antitumor effect against various cancer cells^90,96^. Recently, it was proposed that the aforementioned drug V-9302 targets SLC38A2 and SLC7A5 rather than SLC1A5^87^. A previous study supported this, showing that treatment of *SLC1A5*-knockdown HNSCC cell lines with V-9302 led to marked inhibition of glutamine metabolism, thereby suppressing growth and proliferation both in vitro and in vivo^97^. Therefore, combination therapy with V-9302 and SLC1A5-specific inhibitors may be a promising therapeutic option for some cancers^97^.

### SLC6A14

SLC6A14, also known as amino acid transporter B^0,+^ (ATB^0,+^), maintains a unidirectional influx of glutamine, coupled with 2 Na^+^ and 1 Cl^-^, along a transmembrane gradient^98^. Given the functional role of SLC6A14 in extending the range of amino acid uptake (including glutamine and leucine, both of which are activators of mTORC1), as well as providing substrates for SLC1A5 and SLC7A5, the molecule has attracted much attention^99,100^. Indeed, SLC6A14 is overexpressed in colon, cervical, ER-positive breast, and pancreatic cancer cells and is associated with their proliferation^101–104^. High expression of SLC6A14 in PDAC and CRC cells is closely associated with metastasis and a poor outcome^101,105,106^. Mechanistically, SLC6A14 expression is regulated by the Wnt signaling pathway, and genetic or pharmacological inhibition of the transporter and its downstream effectors suppresses the growth of CRC cells both in vitro and in vivo^102^.

Given that tryptophan is a substrate for SLC6A14, the inhibitor α-methyltryptophan (α-MT) suppresses the growth of SLC6A14-positive breast cancer, PDAC, and CRC cells but not SLC6A14-negative cells^107–109^. Combined treatment of pancreatic cancer cells with gemcitabine and α-MT significantly inhibited proliferation and migration^110^. Although the role of SLC6A14 in cancer cells is becoming clearer, few compounds targeting SLC6A14 have been developed; thus, an effective drug targeting this transporter needs to be developed.

## Mechanisms that induce resistance to glutamine-targeting therapies

**Fig. 2:**
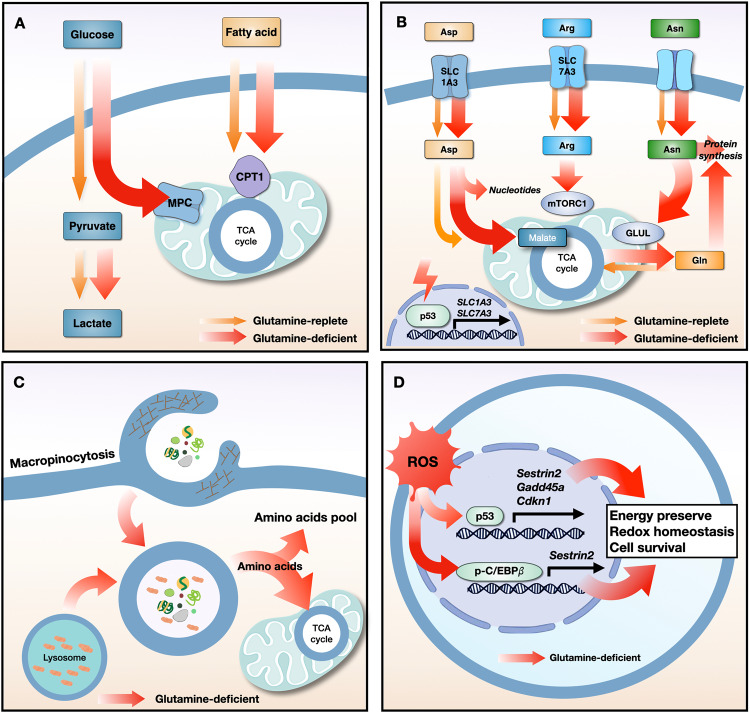
Resistance mechanisms used by cancer cells in response to glutamine starvation. **a** Glutamine starvation induces metabolic flexibility, in which the influx of glucose-derived pyruvate via MPC and fatty acid-derived acyl-CoA via CPT1 into the mitochondria drives TCA cycle activity. **b** Under conditions of glutamine deprivation, the tumor suppressor protein p53 induces the expression of the SLC1A3 and SLC7A2 transporters. Aspartate uptake through SLC1A3 transporters increases the amount of malate, which is a TCA cycle intermediate, leading to an increase in oxidative phosphorylation and glutamine synthesis. Aspartate is used for nucleotide synthesis. Arginine uptake through SLC7A3 transporters restores mTORC1 expression, which is suppressed by glutamine depletion. The high level of intracellular asparagine increases the expression of GLUL proteins, thereby increasing glutamine and protein synthesis. **c** Under conditions of nutrient stress, macropinocytosis internalizes extracellular macromolecules to supply amino acids. Membrane ruffling aids in the uptake of extracellular macromolecules, such as serum albumin, via the formation of macropinosomes. After fusion between macropinosomes and lysosomes, albumin is degraded to supply amino acids to the cytosol and the mitochondrial TCA cycle. **d** Glutamine deprivation increases the expression of p53 and its target genes (*Sestrin2, Gadd45a, and Cdkn1*) and increases the phosphorylation of C/EBPβ and its target gene (*Sestrin2*), all of which maintain energy and redox balance and increase cancer cell survival. MPC, mitochondrial pyruvate carrier; CPT1, carnitine palmitoyltransferase I; TCA, tricarboxylic acid cycle; Asp, aspartate; Arg, arginine; Asn, asparagine; Gln, glutamine; ROS, reactive oxygen species; GLUL, glutamate-ammonia ligase; C/EBPβ, CCAAT/enhancer binding protein β.

Although targeting glutamine metabolism is a promising therapeutic approach, few drugs have been developed. Tumor metabolism is affected by a multitude of microenvironmental factors, including nutrient availability. There are several mechanisms by which cancer cells escape the effects of inhibitors of glutamine metabolism; these include increased metabolic flexibility, uptake of extracellular amino acids via compensatory transporters and macropinocytosis, and expression of nutrient stress-response proteins (Fig. 2).

### Metabolic flexibility

Although glutamine is the primary carbon source for the TCA cycle in some cancer cells, replenishment of TCA cycle intermediates using alternative anaplerotic substrates reduces bioenergetic stress, thereby enabling resistance to inhibition of glutamine metabolism. There are two main anaplerotic flux pathways that feed the citric acid cycle: glutamine flux via glutaminase and glucose flux via pyruvate carboxylase^110^. Upon interruption of glutamine metabolism, glutamine-addicted tumor cells employ compensatory anaplerotic mechanisms via pyruvate carboxylase, which generates the oxaloacetate required to maintain TCA cycle flux; thus, the levels of pyruvate carboxylase can greatly affect the sensitivity of tumor cells to inhibition of glutamine metabolism^111^. In addition, deletion of GLS1 genes from Myc-driven liver tumors upregulates several metabolic compensatory pathways, including glycolysis and aminotransferases^111^. Thus, combined inhibition of glycolytic genes encoding hexokinase II or aminotransferases increases the efficacy of the GLS1 inhibitor CB-839^111^. In contrast, CB-839 showed no antitumor activity in PDAC mouse models due to the use of alternative metabolic pathways (e.g., fatty acid and lipid metabolism) by these cancer cells^45^. Integrated metabolomics and proteomics platforms revealed a marked increase in fatty acid oxidation-related metabolites, as well as proteome changes, in PDAC treated with GLS1 inhibitors, suggesting that treatments should target multiple metabolic pathways to overcome metabolic plasticity^112,113^.

### Extracellular amino acid uptake via compensatory transporters

Amino acids, including aspartate, arginine, and asparagine, are associated with resistance to glutamine depletion. The tumor suppressor protein p53 increases the expression of the aspartate/glutamate transporter SLC1A3 and the arginine transporter SLC7A3 upon glutamine depletion, leading to resistance to treatments that deplete extracellular glutamine^114,115^. Increases in the levels of intracellular aspartate via SLC1A3 contribute to nucleotide synthesis and maintain the electron transport chain and TCA cycle^114^. Although the uptake of arginine by SLC7A3 transporters does not maintain TCA cycle flux under conditions of glutamine depletion, arginine activates mTORC1 and contributes to metabolic adaptation and tumor growth^115^. Uptake of extracellular asparagine prevents the death of glioblastoma cells in response to glutamine depletion by blocking the apoptotic function of a glutamine-induced endoplasmic reticulum stress marker protein, ATF4, and by increasing glutamate-ammonia ligase (GLUL)-mediated glutamine and protein synthesis^116,117^. Thus, blocking amino acid transporters or depleting amino acids such as L-asparaginase may be effective therapeutic strategies to overcome resistance to glutamine withdrawal.

### Macropinocytosis

Macropinocytosis, a nutrient-scavenging pathway, is a compensatory route that supplies amino acids to nutrient-starved cancer cells harboring oncogenic mutations in KRAS or PTEN^118–120^. Experiments using isotope-labeled extracellular proteins show that when supplied with extracellular serum albumin, Ras-transformed cells, which rely on glutamine metabolism to support growth, utilize macropinocytosis to maintain proliferation under glutamine-limiting conditions^120^. More recent studies have shown that regional depletion of glutamine from PDAC tumors stimulates macropinocytosis by activating EGFR/PAK signaling and supplying glutamine via degradation of extracellular proteins in lysosomes^121^. Macropinocytosis also facilitates the survival of hypoxic HCC cells. Thus, HCC cells can internalize extracellular proteins by increasing the expression of a membrane ruffling protein called EH domain-containing protein 2, leading to resistance to glutamine deprivation under hypoxic conditions^122^. Although targeting macropinocytosis could be a key strategy for overcoming resistance to glutamine uptake blockade, further studies are necessary to examine whether macropinocytosis can overcome tumor cell resistance to glutamine antimetabolites or GLS inhibitors that target enzymes involved directly in glutamine metabolism.

### Nutrient stress-response proteins

Limiting glutamine utilization regulates nutrient stress-response proteins and transcription factors. Upon glutamine deprivation, lung cancer cells increase the phosphorylated CCAAT/enhancer binding protein β (p-C/EBPβ)-dependent metabolic protein called Sestrin2 to maintain ATP levels and prevent excessive production of ROS through differential regulation of mTORC1 and mTORC2^123^. Sestrin2-mediated suppression of mTORC1 and mTORC2 activation reprograms lipid metabolism to limit ATP and NADPH consumption, thereby enabling cancer cells to survive under glutamine-depleted conditions. Other studies have shown that ROS production in response to glutamine deprivation increases the expression of p53-dependent genes (*Gadd45a, Cdkn1, and Sestrin2*) via B55α or IKKβ^124,125^. Upregulation of *Gadd45a* and *Cdkn1* induces cell cycle arrest in response to glutamine deprivation, which alleviates oxidative stress and reduces energy consumption^126,127^. Cotargeting proteins involved in glutamine metabolism and the stress response under conditions of glutamine depletion would therefore be a promising therapeutic strategy for overcoming adaptive/resistance mechanisms in cancer cells.

## Effects of targeting glutamine metabolism in the TME

The TME is a complex milieu that surrounds tumor cells, often providing immunosuppressive cover that facilitates immune invasion. Specifically, competition for nutrients or cell-intrinsic programming between cancer cells and immune cells induces nutrient deficiency and metabolic reprogramming of immune cells, leading to modulation of antitumor immunity^128,129^. Given that activation and differentiation of immune cells are coupled to metabolic reprogramming, regulating the metabolic activity of immune cells should be considered in the development of potential strategies that target glutamine metabolism^130,131^.

### Glutamine metabolism in immune cells

Accumulating evidence shows that glutamine is an immunomodulatory nutrient in immune cells. Naïve T cells are metabolically quiescent, undergoing basal levels of glycolysis and glutaminolysis sufficient to maintain minimal biosynthesis; however, T-cell receptor (TCR)-stimulated activation increases the expression of the Myc transcription factor, glutamine transporters (SLC38A1, SLC38A2), and glutaminolysis-related enzymes (GLS, GLUD1, GOT, GPT) to meet bioenergetic and biosynthetic requirements, resulting in T-cell proliferation^130–133^. TCR-induced activation of mTORC1 and metabolic signaling pathways requires SLC1A5-dependent uptake of glutamine; indeed, an SLC1A5-deficient mouse model shows decreased induction of T helper 1 (Th1) and Th17 cells but not Th2, regulatory T (Treg), or CD8(+) T cells, leading to a decrease in proinflammatory T-cell responses^134^. Recent studies have shown that glutamine-derived α-KG regulates the differentiation of CD4(+) T cells into Th1-type effector T cells or Treg cells via DNA methylation and lipid homeostasis^135,136^. Mechanistically, α-KG decreases Treg differentiation by inhibiting FOXP3 and upregulating inflammatory cytokines such as IFN-γ, Tbet, and Rorc, suggesting that Th1-type effector T cells are more dependent on glutaminolysis than Treg cells^136^. Moreover, effector T cells are capable of adapting their metabolism in response to nutrient limitation. Activated T cells rely on glutamine-dependent OXPHOS to maintain energetic homeostasis under energy-related stress (e.g., low glucose levels)^137^.

The antitumor functions of natural killer (NK) cells are upregulated by the expression of c-Myc proteins, which are required for IL-2/IL-12-induced NK cell metabolism and function^138^. Although amino acids are essential for the function of NK cells, their main role in NK cells is the maintenance of signaling (e.g., via c-Myc or mTOR)^139^. Unlike other lymphocyte subsets, glutaminolysis and the TCA cycle do not sustain OXPHOS in activated NK cells. Glutamine withdrawal, but not inhibition of glutaminolysis, results in loss of c-Myc protein, reduced cell growth, and impaired NK cell responses^138^. Consistent with this, receptor-simulated production of IFN-γ by NK cells is not impaired under glutamine-limited conditions^140^.

In macrophages, glutamine metabolism is a critical metabolic pathway for differentiation. Macrophages undergo metabolic switching during differentiation into inflammatory (M1) or anti-inflammatory (M2) phenotypes. Tumor-associated macrophages (TAMs) can exhibit either an antitumor M1-like phenotype or a protumor M2-like phenotype. Glutamine starvation inhibits M2 polarization but not M1 polarization by suppressing UDP-GlcNAc biosynthesis and N-glycosylation of M2-related proteins such as Relmα, CD206, and CD301^141^. Consistent with this, glutaminolysis-derived α-KG promotes M2 activation by increasing fatty acid oxidation and Jmjd3-dependent epigenetic reprogramming of M2-related genes^142^. In contrast to the inhibition of glutaminolysis, pharmacological or genetic targeting of GLUL in macrophages reprograms M2-polarized macrophages to an M1-polarized phenotype^143^. Mechanistically, macrophage-specific inhibition of GLUL leads to accumulation of succinate and HIF-1α via glutamine-dependent γ-aminobutyric acid (GABA) shunting (thereby inhibiting vessel sprouting and metastasis) and via stimulation of T effector cells; however, IL-10-induced expression of GLUL promotes vessel sprouting, immunosuppression, and metastasis^143^.

Given the importance of glutamine metabolism to immune cells, including activated lymphocytes, it is crucial to determine whether blockade of glutamine metabolism in tumor cells hampers anticancer immune responses; the answer may be key to the success of therapeutic strategies targeting glutamine metabolism.

#### Glutamine blockade in the TME

The metabolism of cancer cells and immune cells in the TME is regulated by cell-intrinsic programs through mTORC1 signaling^128^. PET tracers showed that cancer cells rely heavily on glutamine uptake via mTORC1 signaling, while myeloid cells in the TME are more dependent on glucose, as are T cells and cancer cells (but to a lesser extent)^128^. Although administration of V-9302 to decrease glutamine availability increases glucose uptake by cancer cells and immune cells in allograft models, the growth of tumors harboring tumor-infiltrating Tregs, CD8(+) T cells, and NK cells is suppressed^128^. Given that cancer cells are much more dependent on glutamine than immune cells and that V-9302 does not impair CD8(+) T-cell viability and activation^83^, pharmacological inhibitors of SLC1A5 might have high therapeutic potential.

**Fig. 3:**
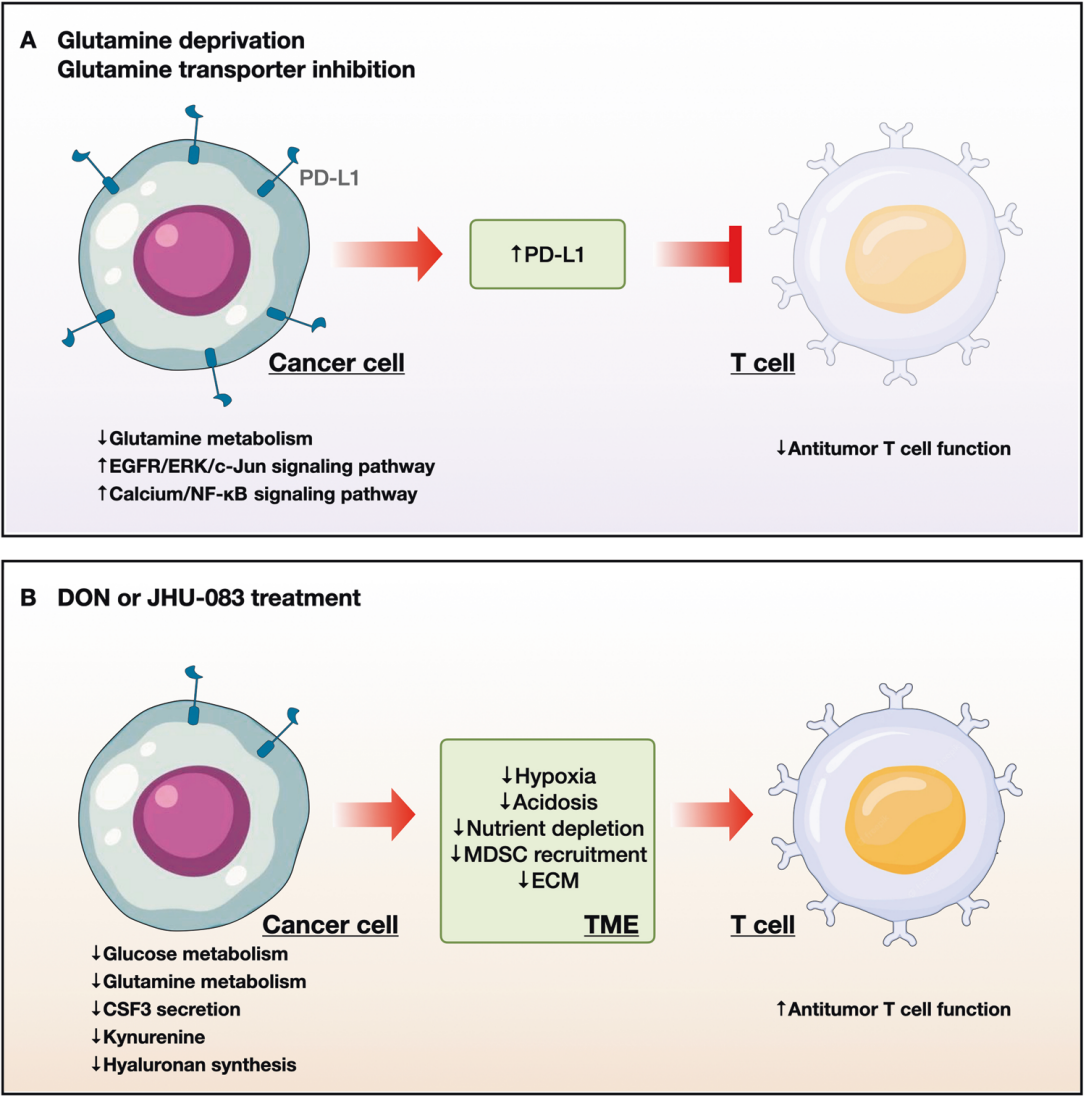
T-cell-mediated immune responses to glutamine-targeted treatment in cancer cells. **a** Glutamine deprivation and transporter inhibition decrease glutamine metabolism, thereby boosting EGFR/ERK/c-Jun signaling and calcium/NF-kB signaling, leading to upregulation of PD-L1. PD-L1 suppresses antitumor immune responses by blocking T-cell activation in the tumor microenvironment. **b** Treatment with glutamine analogs, including DON and JHU-083, decreases glucose and glutamine metabolism, leading to inhibition of tumor growth via a decrease in hypoxia, acidosis, and nutrient depletion in the tumor microenvironment. Furthermore, DON decreases the recruitment of MDSCs by suppressing the secretion of CSF3 by tumor cells and blocking the production of the immunosuppressive metabolite kynurenine; this inhibits the synthesis of the hyaluronan-rich ECM, resulting in the activation and infiltration of T cells. PD-L1, programmed death-ligand 1; CSF3, colony stimulating factor 3; MDSC, myeloid-derived suppressor cell; ECM, extracellular matrix.

Accumulating evidence shows that inhibitors of glutamine metabolism, such as V-9302, JHU-083, and CB-839, elicit stronger antitumor effects when used in combination with immune checkpoint inhibitors^144–146^ (Fig. 3). In a previous study, we showed that V-9302 induces the expression of PD-L1 by tumor cells and augments immune evasion in synergistic murine models^58^. Mechanistically, glutamine limitation decreases GSH levels and sarco/endoplasmic reticulum Ca^2+^-ATPase (SERCA) glutathionylation, resulting in reduced SERCA activity^58^. Upregulation of cytosolic Ca^2+^ activates calcium/calmodulin-dependent protein kinase II (CAMKII), leading to aberrant NF-κB signaling and downstream expression of PD-L1^58^. Therefore, agents that target glutamine utilization may, when used in combination with an anti-PD-L1 antibody, boost antitumor immunity^58^. Similar results were reported for several tumors^147–150^. Glutamine starvation increases the expression of PD-L1 in RCC and bladder cancer cells via the EGFR/ERK/c-Jun signaling pathway^147,148^. Furthermore, bladder tumors in mice supplemented with glutamine showed lower PD-L1 levels than control tumors^148^. In natural killer T-cell lymphoma (NKTCL), blocking SLC1A1-mediated glutamine addiction in tumor cells induced PD-L1 expression and inhibited CD8(+) T-cell activity^149^. As a therapeutic option, combined treatment with asparaginase and an anti-PD-1 antibody could be useful because glutamine-addicted cells are sensitive to asparaginase^149^.

In contrast to PD-L1-mediated T-cell dysfunction induced by glutamine limitation, DON and its prodrug JHU-083 skew CD8(+) T cells toward a highly activated, persistent, and proliferative phenotype, thereby facilitating immunogenic cancer cell death^58,149,150^ (Fig. 3). DON treatment activates AMPK in cancer cells by increasing the AMP/ATP ratio and decreasing the expression of c-Myc proteins, thereby inhibiting glycolytic metabolism and suppressing cancer cell growth in vitro^145^. However, CD8(+) T cells in vitro overcome metabolic stress through glucose-dependent anaplerosis and acetate catabolism. Another study showed that in JHU-083-treated cancer cell allograft models, an increase in nutrient levels and oxygen and a decrease in the acidity of the TME resulted in T-cell-mediated tumor suppression^145^, whereas another study demonstrated the effects of JHU-083 on myeloid-derived suppressor cells (MDSCs) and TAMs^150^. JHU-083 markedly suppresses the recruitment of MDSCs by decreasing tumor-derived CSF3 levels via degradation of C/EBPβ, which in turn increases the numbers of proinflammatory TAMs and enhances antigen presentation to CD8(+) T cells, resulting in stronger T-cell responses^150^. In addition, JHU-083 reduces the expression of IDO (an enzyme that mediates tryptophan metabolism) by inhibiting the phosphorylation of STAT1 and STAT3 in tumor cells, MDSCs, and TAMs, thereby decreasing the kynurenine/tryptophan ratio and enhancing the functions of antitumor T cells^150^. Treatment of PDAC with DON decreases uridine diphosphate N-acetylglucosamine (UDP-GlcNAc) levels, which affects synthesis of the hyaluronan-rich extracellular matrix (ECM); this suggests that glutamine inhibitors such as DON could deplete the ECM and allow infiltration of tumors by CD8(+) T cells^151^.

Two compounds (V-9302 and JHU-083) that target SLC1A5 and glutamine metabolism in allograft models elicited different T cell-mediated immune responses; however, because these compounds do not reduce the activation or viability of CD8(+) T cells^83,145^, combined immunotherapy significantly improves their antitumor effects.

## Conclusion

Glutamine metabolism plays a central role in regulating uncontrolled tumor growth by modulating bioenergetic and redox homeostasis and by serving as a precursor for the synthesis of biomass. Although targeting glutamine metabolism is a promising strategy for cancer therapy, there are many hurdles to be overcome before we develop a clinically effective drug. Metabolic flexibility or adaptation by cancer cells, as well as reduced antitumor immunity, may be unwanted consequences of inhibiting glutamine metabolism. A comprehensive understanding of the TME is of the utmost importance because it provides valuable insights into pathways that could be targeted by novel metabolic therapies for advanced or drug-resistant cancers.
